# Protective Effect of Marjoram Against Letrozole-Induced Ovarian Damage in Rats with Polycystic Ovarian Syndrome Entails Activation of Nrf2 and Suppression of NF-κB

**DOI:** 10.3390/ph18091291

**Published:** 2025-08-28

**Authors:** Laila Naif Al-Harbi, Sahar Abdulaziz ALSedairy, Ghedeir M. Alshammari, Manal Abdulaziz Binobead, Shaista Arzoo

**Affiliations:** Department of Food Sciences and Nutrition, College of Food Science and Agriculture, King Saud University, Riyadh 11451, Saudi Arabia; ssudairy@ksu.edu.sa (S.A.A.); aghedeir@ksu.edu.sa (G.M.A.); mbinobead@ksu.edu.sa (M.A.B.); sarzoo@ksu.edu.sa (S.A.)

**Keywords:** polycystic ovary syndrome, oxidative stress, inflammation, Nrf2

## Abstract

**Objectives**: This study aimed to evaluate marjoram’s ameliorative effects in a letrozole-induced PCOS rat model and to explore its mechanism of action, focusing on Nrf2 activation and NF-κB suppression in ovarian tissue. **Methods**: In this study, PCOS was induced by the oral administration of letrozole (1 mg/kg/day) for 21 days. Rats were then divided into six groups: control (0.5% CMC), letrozole, letrozole + metformin (2 mg/100 g), and letrozole + MRJ extract (20, 40, or 60 mg/kg). All groups received oral treatment for 21 days. Biochemical analysis was performed using serum and plasma; while ovarian tissue homogenate was used for antioxidant enzymes and inflammatory and apoptosis biomarkers. **Results**: The letrozole-treated animals exhibited significant increases in final body weights, as well as ovary length and weight. In terms of biochemical parameters, there were significant increases in fasting blood glucose and insulin, HOMA-IR, and serum levels of cholesterol, triglycerides (TGs), and LDL-c and a decrease in HDL levels. Concerning the hormonal profile, testosterone and LH levels were significantly elevated while a notable decrease in FSH and estradiol levels was observed. Similarly, letrozole-treated rats showed significantly elevated levels of MDA and many other inflammatory mediators such as IL-6, TNF-α, and ICAM-1. A significant increase in the markers of intrinsic cell apoptosis, such as Bax and caspase-3, and the reduced levels of Bcl-2 and antioxidant mediators, including GSH, SOD, and HO-1, as well as mRNA and nuclear expression of Nrf2, compared to control rats, have been reported. The ovaries of the rats with PCOS treated with metformin and MRJ (60 mg/kg) showed the most significant improvements. Similarly, TEM also demonstrated a dose-dependent ameliorating effect. **Conclusions**: The current study highlights marjoram’s protective effect against letrozole-induced ovarian damage in rats with polycystic ovarian syndrome, suggesting its potential as a complementary and therapeutic agent for managing PCOS.

## 1. Introduction

Polycystic ovary syndrome (PCOS) is a heterogeneous and chronic endocrine disorder, affecting about 5–15% of women in their reproductive age [1,2,3,4]. The main characteristics of PCOS are hyperandrogenism, ovarian cysts, and polycystic ovarian changes. PCOS itself, regardless of infertility status, is also associated with the metabolic health of patients [5]. Although its exact pathophysiology is still vague, numerous studies have advocated that various factors such as genetics, androgen excess, insulin resistance (IR), obesity, inflammation, and oxidative stress (OS) may be involved in the pathogenesis of this disease [6,7,8,9,10]. Moreover, the prevalence of metabolic disorders such as type 2 diabetes (T2D), obesity, cardiovascular disease, and dyslipidemia is also higher in women with PCOS [6,7,11,12,13]. Due to hyperandrogenism, it leads to insulin resistance and hyperglycemia, which in turn lead to reactive oxidative stress formation, inflammation, and abdominal adiposity [6]. These factors might play a significant role in the pathology and signaling of PCOS [14]. Hyperinsulinemia, considered as insulin resistance, enhances the production and release of androgen by theca cells in the ovaries and reduces the hepatic production of sex-hormone-binding globulin. These effects lead to the development of the hyperandrogenism characteristics of PCOS. Additionally, in PCOS, IR is exacerbated due to OS, which leads to dysfunction in the pathway of insulin signaling [9,15]. Inflammation also plays an essential role in its pathogenesis [16]. Overexpression of inflammatory markers, including tumor necrosis factor-alpha (TNF-α), interleukins (IL) such as IL-6, IL-1β, and IL-18, and OS are documented to play vital roles in the pathophysiology process of various human diseases including PCOS, affecting reproduction. Biomarkers of OS and altered antioxidant defenses have been demonstrated in women with PCOS. Nuclear factor erythroid 2-related factor 2 (Nrf2) is a key transcriptional factor and considered as a master regulator of cellular responses against OS that induces antioxidants [17]. Enzymatic antioxidant induction via Nrf2 plays a critical role in the management of OS in PCOS.

The medical management of PCOS is aimed to manage the clinical symptoms, and currently, metformin is one of the most popular and widely used medications for its management. In addition, it is used to manage T2D as it acts as an anti-hyperglycemic agent that improves IR [18]. In addition to its effect on improving IR, metformin has been widely demonstrated to play a significant role in inducing ovulation in women with PCOS by decreasing ovarian androgen production [19,20]. Moreover, it has been associated with metabolic benefits that may involve in weight and lipid level management. However, the most common side effects of metformin are gastrointestinal such as abdominal pain, indigestion, nausea, diarrhea, and flatulence [21,22]. Apart from these gastrointestinal side effects, numerous studies have observed an association between metformin use and low vitamin B12 levels [23,24]. Although extremely rare, metformin-associated lactic acidosis (MaLA) is considered to be a lethal adverse effect [25]. A previous study has shown that oral doses of metformin (30 mg/kg) and glibenclamide (5 mg/kg) over 21 days induced testicular OS and histopathological damage in rats [26]. Another study, performed in rats with PCOS, reported that a polyherbal formulation given at a dose of 400 mg/kg is more effective than metformin in improving the symptoms or conditions caused by PCOS [27]. Consequently, the use of metformin appears to be limited in improving reproductive outcomes in women with PCOS.

In the past decade, women have shown a growing interest in using herbal medicines as forms of complementary and alternative medicines [28,29,30]. Herbal medicines are the “extract of whole plant or any part of the plant that shows a major therapeutic effect and fewer side effects than conventional medicine and their effect is consequent of their active ingredients” [31]. Previous studies have reported that many of herbal ingredients, such as *Camellia sinensis* [32] and *Cinnamon* [33], have been used to reduce the symptoms of PCOS. In their study, Alaee et al. have reported that spearmint extract may alleviate key PCOS-related symptoms by reducing body weight and testosterone levels while improving ovarian morphology [34]. In their review, Kwon et al. [35] have concluded that herbal medicines improve the estrous cycle, stabilize the female hormones and reduce the male hormones, and improve lipid metabolism and IR. However, the effectiveness of herbal treatment is quite uncertain due to their short duration of studies, lack of comprehensive phytochemical characterization, standardization, and bioavailability, making it more difficult to determine the active constituents responsible for therapeutic effects, and lack of clinical trials in women with PCOS.

Marjoram (*Origanum majorana* L.), a perennial herb from the mint family, which is characterized for its flavor, is commonly used to treat various ailments, including diabetes and neurological and digestive issues. The bioactive compounds in marjoram have been shown to have therapeutic potential for metabolic and reproductive disorders, including PCOS [36]. In traditional medicine, marjoram therapy has been found to be related to women with menstruation problems and PCOS [37,38]. In addition, it has been reported that marjoram was found to improve insulin sensitivity and reduce the adrenal androgen levels [39]. A study by Rababa’h et al. [21] showed that the marjoram-treated group had significantly decreased the level of estradiol and improved insulin sensitivity in the PCOS (DHEA-induced) rats. Moreover, marjoram has many biological activities as anti-inflammatory [40] and antioxidant [41]. As a result of these promising findings, further investigation into the underlying mechanisms of marjoram’s action in conditions like PCOS is warranted. Therefore, using a rat model of letrozole-induced PCOS, this study aimed to assess the potential ameliorative benefits of marjoram in the treatment of PCOS, as well as its mechanism of action by targeting its effect on ovarian rats with polycystic ovarian syndrome via the activation of Nrf2 and the suppression of NF-κB.

## 2. Results

### 2.1. Quantitative Analysis of Marjoram Methanolic Extract

**Figure 1:**
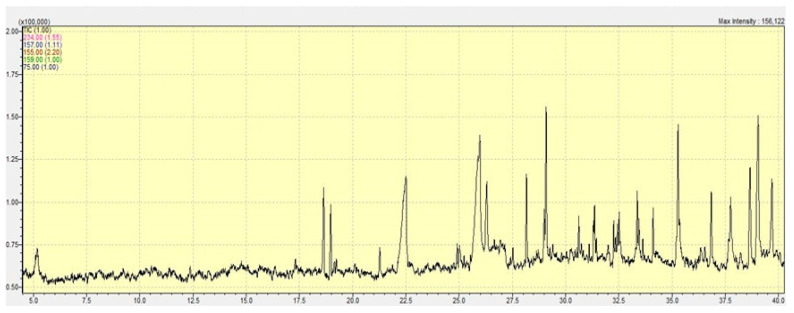
GC-MS profile of methanolic leaf extracts of *Origanum majorana* L.

In the present study, the gas chromatography–mass spectrometry (GC–MS) analysis of *Origanum majorana* L. sample revealed the presence of several compounds with varying retention times, molecular weights, and concentrations (%). The identified compounds are summarized in Figure 1 and Table 1. It has been observed that 1,1,6-trimethyl-3-methylene-2-(3,6,9,13-tetramethyl-6-ethenye-10,14-dimethylene-pentadec-4-enyl) cyclohexane exhibited the highest relative abundance (4.74%) followed closely by Henicosane-6,8-dione (4.71%). Other prominent compounds including Heptacosane-6,8-dione, Stigmasterol, and Dodecanoic acid were present in the least amount. The retention times ranged from 12.35 min to 39.05 min, indicating the presence of both low and high polarity and volatility of compounds within the sample.

### 2.2. Body and Ovary Weight

The final body weights, as well as the right and the left ovary lengths and weights, were significantly increased in letrozole-treated rats compared to all other groups of rats. The final body weight, right ovary weight and length, and left ovary weight and length were all decreased in letrozole-treated animals post-metformin for all tested doses of MRJ. However, the reduction in these markers in letrozole + MRJ was dose-dependent, with a maximum effect seen at the highest dose (60 mg/kg). Difference in the final body weights, as well as ovary weights and lengths, were insignificant, when the control group was compared with letrozole + MRJ-treated rats (60 mg/kg) and when the letrozole + Met-treated rats were compared with the letrozole + MRJ-treated rats (20 mg/kg) (Table 2).

### 2.3. Fasting Blood Glucose (FBG), Fasting Blood Insulin (FBI), and HOMA-IR

There was a significant upsurge in fasting blood glucose and insulin levels and HOMA-IR as a marker IR in letrozole-treated animals compared to control rats. As expected, a significant reduction in the levels of all these markers in the metformin-treated animals has been noted. However, no significant differences were found between letrozole-treated rats and all letrozole + MRJ-treated rats at any dose (Table 3).

### 2.4. Lipid Profile

Table 4 showed the effect of different treatments of MRJ on the lipid profile in all experimental groups of rats. It has been observed that cholesterol, TG, and LDL-c serum levels increased significantly, whereas, in contrast, the serum levels of HDL were significantly reduced in letrozole-treated rats when compared to rats of the control group. All of these lipid levels were reversed with Met and MRJ treatment as compared to letrozole treatment. There was a partial improvement in the levels of these lipids in letrozole + Met treatment. A reduction in the levels of TGs, cholesterol, and LDL-c parallel to the dose–response increase in HDL-c levels was seen in letrozole + MRJ-treated animals (20, 40, and 60 mg/kg). Insignificant differences (except for cholesterol) were observed in the serum levels of all of these markers between the control and the letrozole + MRJ (60 mg/kg) group.

### 2.5. The Levels of Major Sex Hormones in the Serum of All Groups of Rats

**Figure 2:**
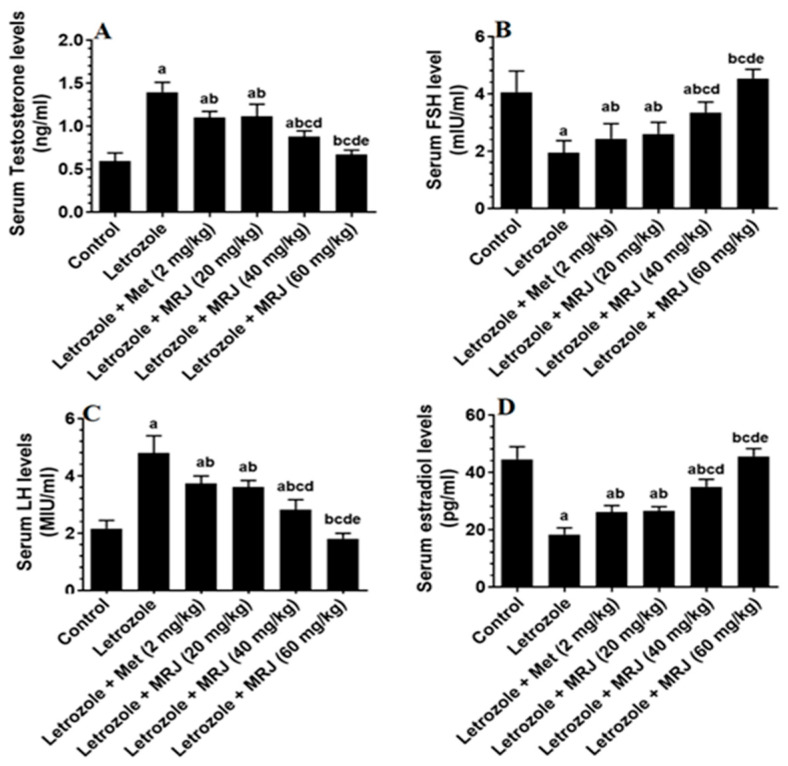
The levels of major sex hormones ((**A**) Testosterone; (**B**) FSH; (**C**) LH and (**D**) Estradiol) in the serum of all groups of rats. Data are presented as means ± SD for n = 7 rats/group. Values were considered significantly different at *p* < 0.05. (a): Significantly differed from the control group; (b): significantly differed from letrozole-treated rats; (c): significantly differed from letrozole + metformin (Met)-treated rats; (d): significantly differed from letrozole + MRJ-treated animals (20 mg/kg); and (e): significantly differed from letrozole + MRJ-treated animals (40 mg/kg). Note: MRJ: *Origanum majorana*; Met: metformin, FSH: follicle-stimulating hormone; LH: luteinizing hormone.

As depicted in Figure 2, letrozole treatment significantly increased serum testosterone and LH and decreased FSH and estradiol levels compared to controls (Figure 2). These changes were partially reversed by all treatments. A reduction in testosterone and LH levels, along with an increase in FSH and estradiol levels, was observed in the MRJ-treated group. These changes were significantly different from those in the letrozole-treated rats but not significantly different when compared to the control group.

### 2.6. mRNA and Nuclear Levels of NF-κB and Nrf2 in Rat Ovaries

**Figure 3:**
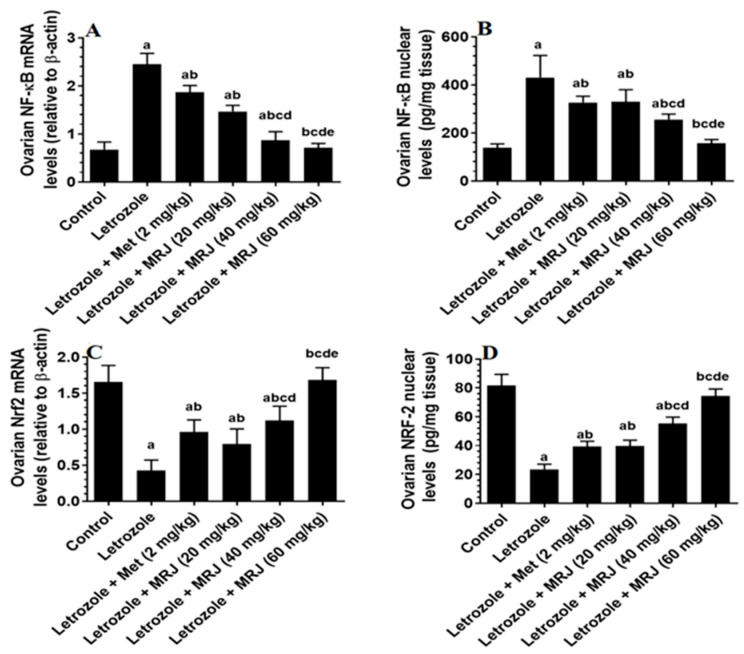
mRNA and nuclear levels of NF-κB ((**A**) NF-κB mRNA; (**B**) NF-κB nuclear levels) and Nrf2 ((**C**) Nrf2 mRNA; (**D**) Nrf2 nuclear levels) in the ovaries of all groups of rats. Data are presented as means ± SD for n = 7 rats/group. Values were considered significantly different at *p* < 0.05. (a): Significantly differed from the control group; (b): significantly differed from letrozole-treated rats; (c): significantly differed from letrozole + metformin (Met)-treated rats; (d): significantly differed from letrozole + MRJ-treated animals (20 mg/kg); and (e): significantly differed from letrozole + MRJ-treated animals (40 mg/kg). Note: MRJ: *Origanum majorana*; Met: metformin; Nrf2: Nuclear Factor Erythroid 2-Related Factor 2; NF-κB: Nuclear Factor-kappa B.

The ovaries of rats treated with letrozole showed significantly higher mRNA and nuclear levels of NF-κB than control rats, but mRNA levels and nuclear levels of Nrf2 were significantly lower (Figure 3). These were reversed after treatment with metformin or MRJ at all tested doses (20, 40, and 60 mg). Among all doses, only treatment with the highest doses induced normal mRNA and nuclear levels of Nrf2 and NF-κB in the ovaries of letrozole-treated rats, which were not significantly different as compared to control rats (Figure 3). In addition, the mRNA and nuclear levels of Nrf2 were considerably higher, and the mRNA and nuclear levels of NF-κB were significantly lower as compared to their levels in the letrozole + MRJ (20 and 40 mg/kg)-treated rats (Figure 3).

### 2.7. Levels of Some Inflammatory Mediators in Ovaries of All Experimental Groups

**Figure 4:**
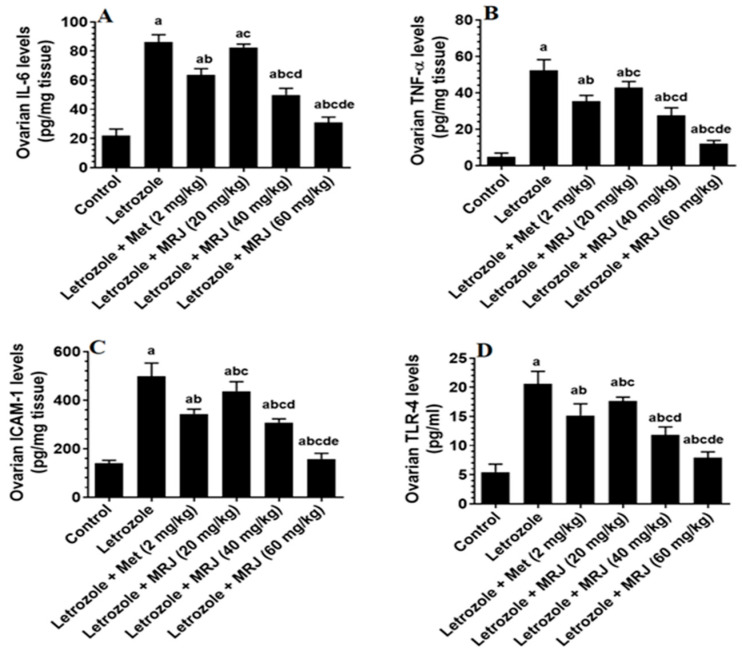
The levels of some inflammatory mediators in the ((**A**) IL-6; (**B**) TNF-α; (**C**) ICAM-1; and (**D**) TLR-4) ovaries of all experimental groups. Data are presented as means ± SD for n = 7 rats/group. Values were considered significantly different at *p* < 0.05. (a): Significantly differed from the control group; (b): significantly differed from letrozole-treated rats; (c): significantly differed from letrozole + metformin (Met)-treated rats; (d): significantly differed from letrozole + MRJ-treated animals (20 mg/kg); and (e): significantly differed from letrozole + MRJ-treated animals (40 mg/kg). Note: MRJ: *Origanum majorana*; Met: metformin; IL- interleukins; TNF-α: tumor necrosis factor-α; ICAM-1: intracellular cell adhesion molecule-1; TLR4: toll-like receptor.

Ovarian level of TNF-α, IL-6, TLR4, and ICAM-1 increased significantly in letrozole-treated animals, while a significant reduction in the level of these inflammatory mediators was observed in the ovaries of letrozole + Met-treated and letrozole + MRJ (20, 40, and 60 mg/kg)-treated rats when compared with letrozole-treated animals (Figure 4). The levels of all these inflammatory factors decreased progressively and significantly in a dose-dependent manner. The present study did not observe any significant difference in these levels between the control and letrozole + MRJ (60 mg/kg) groups (Figure 4). However, in letrozole + Met-treated rats, levels of this biochemical endpoint remained significantly higher than in control rats, but significantly lower than in the letrozole + MRJ (20 or 40 mg/kg)-treated rats (Figure 4).

### 2.8. Levels of Some Antioxidant Mediators in Ovaries of All Experimental Groups

**Figure 5:**
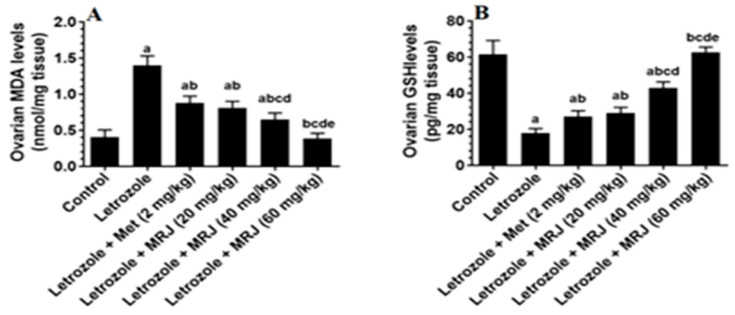

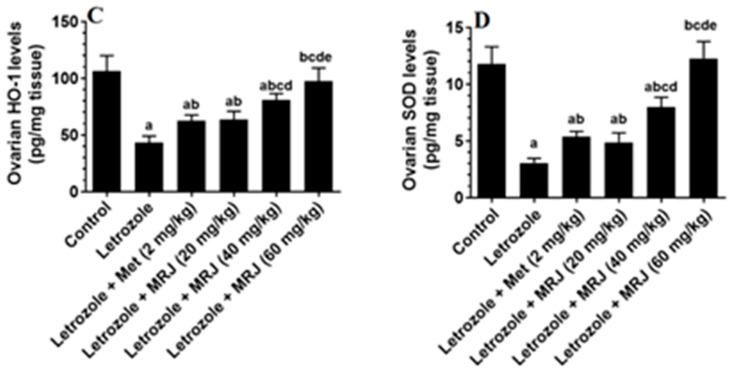
The levels of some antioxidant mediators ((**A**) MDA; (**B**) GSH; (**C**) HO-1; and (**D**) SOD) in the ovaries of all experimental groups. Data are presented as means ± SD for n = 7 rats/group. Values were considered significantly different at *p* < 0.05. (a): Significantly differed from the control group; (b): significantly differed in letrozole-treated rats; (c): significantly differed from letrozole + metformin (Met)-treated rats; (d): significantly differed from letrozole + MRJ-treated animals (20 mg/kg); and (e): significantly differed from letrozole + MRJ-treated animals (40 mg/kg). Note: MRJ: *Origanum majorana*; Met: metformin; MDA: malondialdehyde; GSH: total glutathione; HO-1: heme-oxygenase-1; SOD: superoxide dismutase.

In comparison to control rats, letrozole-treated animals’ ovaries showed a considerable decrease in GSH, SOD, and HO-1 levels and a large increase in MDA levels (Figure 5), while these levels reversed in the ovaries of letrozole-treated rats that received metformin or MRJ (20, 40, and 60 mg/kg) as compared to those who received the vehicle (Figure 5). The result depicts that, in comparison to all other treatments, letrozole + MRJ (60 mg/kg) produced the greatest improvement in all of these indicators’ levels, and in these rats, the levels of all of these markers did not differ substantially from those of the control group (Figure 5).

### 2.9. Levels of Some Markers of Intrinsic Cell Apoptosis in Ovaries of All Experimental Groups

**Figure 6:**
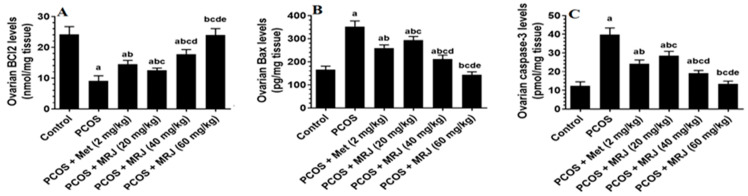
The levels of some markers of intrinsic cell apoptosis ((**A**) Bcl-2; (**B**) Bax; and (**C**) Capase-3) in the ovaries of all experimental groups. Data are presented as means ± SD for n = 7 rats/group. Values were considered significantly different at *p* < 0.05. (a): Significantly differed from the control group; (b): significantly differed from letrozole-treated rats; (c): significantly differed from letrozole + metformin (Met)-treated rats; (d): significantly differed from letrozole + MRJ-treated animals (20 mg/kg); and (e): significantly differed from letrozole + MRJ-treated animals (40 mg/kg). Note: MRJ: *Origanum majorana*; Met: metformin; Bcl-2: B-cell lymphoma 2; Bax: Bcl-2-associated protein x.

There was a significant rise in Bax and caspase-3 levels with a significant concomitant decrease in the ovarian Bcl-2 levels in letrozole-treated animals as compared to control rats, which were reversed with all treatments (Figure 6). Comparing letrozole + Met, letrozole + MRJ (20 mg/kg), and letrozole + MRJ (40 mg/kg) rats with control rats, the levels of Bcl-2 were significantly lower and the levels of Bax and caspase-3 were significantly higher (Figure 6). In addition, there was a significant progressive variation in the levels of these markers with increasing doses of MRJ. Of note, the levels of Bcl-2, Bax, and caspase-3 were not significantly different in the ovaries of letrozole + MRJ (60 mg/kg)-treated rats when compared to these levels measured in the ovaries of control rats (Figure 6).

### 2.10. Ultrastructural Findings

**Figure 7:**
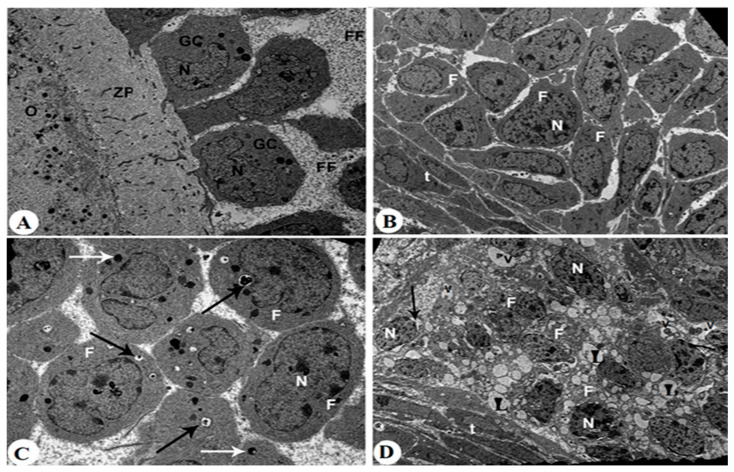
Transmission electron micrographs (TEMs) are taken for all control (**A**,**B**) and letrozole-treated rats (**C**,**D**). **Photomicrograph A**: showing an early intact antral follicle that has spaces between adjacent granulosa cells (GCs) filled with follicular fluid (FF). The granulosa cells surrounding the oocyte (O) through zona pellucid (ZP) are seen, ×5000. **Photomicrograph B**: showing a secondary follicle with several layers of follicular cells (F) surrounded by the theca layer (t). In addition, each of these follicular cells contained an intact nucleus (N), ×5000. **Photomicrograph C**: showing the secondary follicular cells containing an increased number of phagocytic vacuoles (black arrows) and electron-dense granules (white arrows), ×5000. **Photomicrograph D**: showing extreme cytoplasmic degeneration in the secondary follicular cells that appeared to contain plenty of lipid vacuoles (V) and pyknotic nuclei (N) as well as a dilated nuclear membrane (arrow), ×5000.

**Figure 8:**
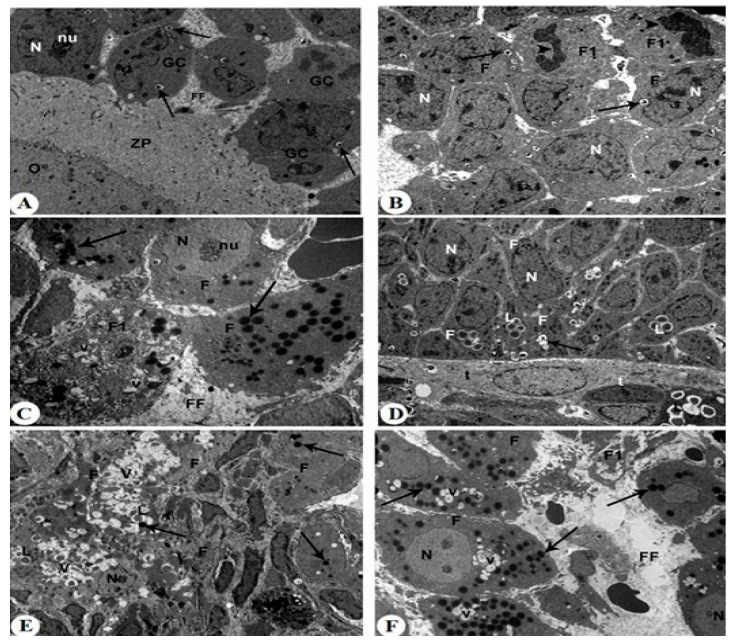
Transmission electron micrographs (TEMs) are taken for all letrozole + Met-treated rats (**A**–**C**) and letrozole + MRJ (20 mg/kg)-treated rats (**D**–**F**). **Photomicrograph A:** showing an increased number of phagocytic vacuoles (Arrow) in the antral follicle. However, almost intact granulosa cells (GCs), follicular fluid (FF), and granulosa cells surrounding the oocyte (O) through zona pellucid (ZP) are seen, ×5000. **Photomicrograph B:** showing two follicular cells (F1) in the state of mitotic division (head arrows) and an increased number of small phagocytic vacuoles (Arrow), × 5000. **Photomicrograph C:** showing a damaged and degenerated secondary follicular cell (F1) containing phagocytic vacuoles (V), euchromatic nuclei (N), segregation of nucleolus (nu), and a large number of electron-dense granules (arrows), ×5000. **Photomicrograph D:** showing severe cytoplasmic degeneration in follicular cells (F), which still bearing plenty of vacuoles (V) and pyknotic nuclei (N), a dilated nuclear membrane (Arrow), and lipid droplets (L), surrounded by the theca layer (t), ×5000. **Photomicrograph E:** showing severe degeneration in the secondary follicular cells (F) with the existence of a high number of phagocytic (V) and lipid vacuoles (L), as well as shrunk nucleus (N) and few electron-dense granules (arrows), ×5000. **Photomicrograph F:** showing a damaged and degenerated secondary follicular cell (F1), euchromatic nuclei (N) with segregation of nucleolus (nu), an increased number of lipid and phagocytic vacuoles (V), and a large number of electron-dense granules (arrows), and an increased number of lipid and phagocytic vacuoles (V), ×5000.

**Figure 9:**
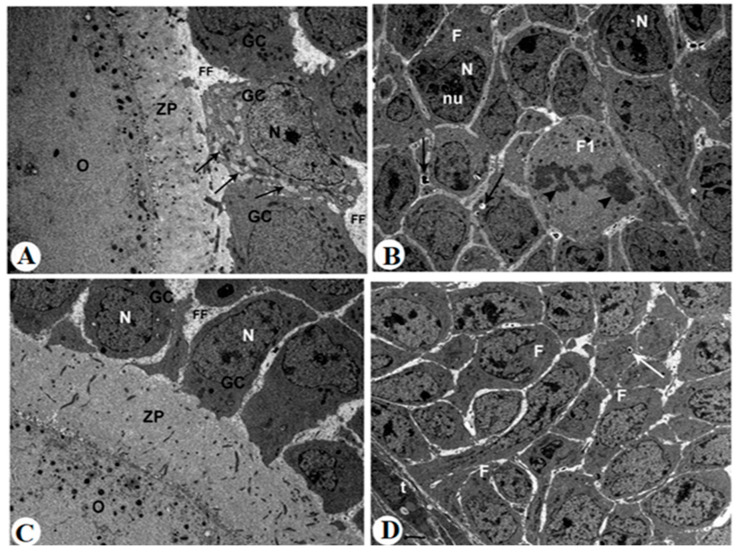
Transmission electron micrographs (TEMs) are taken for letrozole + MRJ (40 mg/kg)-treated rats (**A**,**B**) and letrozole + MRJ (60 mg/kg)-treated rats (**C**,**D**). **Photomicrograph A:** showing an early intact antral follicle that has spaces between adjacent granulosa cells (GCs) filled with follicular fluid (FF). The granulosa cells surrounding the oocyte (O) through zona pellucid (ZP) are intact. However, even the majority of follicles are almost normal, some of them show a high number of dilated vacuoles (Arrows), ×5000. **Photomicrograph B:** showing an extremely dilated and damaged secondary follicular cell (F1) in its mitotic division state (Head arrows), and few small phagocytic vacuoles (Arrow). Other follicles (F) show degeneration in the nuclei (N) with segregation of nucleolus (nu), ×5000. **Photomicrograph C:** showing a normal antral follicle with normal GC, ZP, and FF, ×5000. **Photomicrograph D:** showing normal secondary follicles with just very few numbers of phagocytic vacuoles (arrows), ×5000.

Ovaries from control rats exhibited intact antral and secondary follicles with normal nuclear morphology, characterized by well-defined nuclear membranes and uniformly distributed chromatin (Figure 7A,B). The cytoplasm of granulosa and follicular cells appeared intact, with minimal vacuolation and abundant organelles. Follicular fluid (FF) was clearly observed filling the spaces between granulosa cells and surrounding the oocyte within the zona pellucida. In contrast, ovaries from letrozole-treated rats showed marked nuclear alterations, including dilated nuclear membranes, pyknotic nuclei, and nucleolar segregation (Figure 7C,D). Cytoplasmic changes consisted of numerous phagocytic and lipid vacuoles along with electron-dense granules, indicating severe degeneration. Follicular fluid appeared reduced and irregular, often associated with disorganized granulosa cell layers. In letrozole + metformin-treated rats, nuclear damage was less pronounced, with the partial preservation of nuclear integrity (Figure 8A–C). Cytoplasmic changes were moderate, showing limited vacuolation, and follicular fluid distribution was partially restored compared to the letrozole group. Treatment with MRJ at 20 mg/kg still showed significant nuclear condensation (Figure 8D–F). Cytoplasmic degeneration persisted, with abundant vacuoles present, and follicular fluid remained unevenly distributed. MRJ at 40 mg/kg improved nuclear architecture, reduced cytoplasmic degeneration, and partially normalized follicular fluid appearance (Figure 9A,B). The highest dose of MRJ (60 mg/kg) produced near-normal nuclear morphology, minimal cytoplasmic changes, and a uniform distribution of follicular fluid, resulting in follicles that closely resembled the control group (Figure 9C,D).

## 3. Discussion

PCOS is one of the most common prevalent metabolic dysfunctions associated with an increased risk of insulin resistance affecting women in their reproductive age. In this study, the methanolic extract of marjoram was examined by using GC-MS analysis to provide a comprehensive profile of its biologically active compounds. The methanolic extract of *Origanum majorana* contains various bioactive compounds having potential diverse pharmacological activities. 1,1,6-trimethyl-3-methylene-2-(3,6,9,13-tetramethyl-6-ethenyl-10,14-dimethylene-pentadec-4-enyl)cyclohexane is notable for its antimicrobial, anticancer, antiarthritic, anti-inflammatory, and antiviral properties [42,43]. Henicosane-6,8-dione and Heptacosane-6,8-dione were the other prominent compounds noted in marjoram. A previous study has demonstrated that Heneicosane is an effective antimicrobial against *Aspergillus fumigatus* and *Streptococcus pneumoniae* [44]. Vitamin E has antioxidant, anti-aging, hypoglycemic, anti-inflammatory, anticancer, and anticoronary properties [45]. According to Jeruto et al. [46], flavonoids, terpenoids, and steroids possess a protective effect on red blood cells due to their antioxidant properties. In LPS-induced acute lung injury, 5-hydroxymethylfurfural (5-HMF) has been shown to possess anti-inflammatory and protective effects. Its therapeutic action against NLRP3 inflammasome-related inflammatory disorders is mediated through the inhibition of endoplasmic reticulum stress [47].

In the present study, the effects of different doses of methanolic extract of marjoram against letrozole-induced ovarian damage in female rats were evaluated. This study focused on changes in hormonal profile, body and ovarian weight, insulin sensitivity, lipid profile, and ovarian inflammation. Furthermore, the extract’s ability to improve antioxidant responses through the activation of Nrf2 and the suppression of NF-κB was assessed as a potential mechanism for protecting the ovary against PCOS-induced damage. The reproductive characteristics of PCOS in female Wistar rats were successfully produced by the oral administration of letrozole (1 mg/kg, per orally, p.o.) for 21 days. Previous studies reported the efficacy of letrozole in establishing and inducing the PCOS state and the metabolic phenotypes of female rat mimicking human polycystic ovary syndrome [48,49]. Letrozole-treated rats in the PCOS rat model gained body weight and their ovary weight also increased compared with the control. This finding is in agreement with that of a previously reported study [50,51]. Importantly, an increase in body weight and the weight of reproductive organs are primarily attributed to the effect of letrozole on the block conversion of androgens to estrogens, leading to hyperandrogenism [52]. Our data showed that the ovary weight and length were decreased in letrozole-treated animals following treatment with either metformin or any of the tested doses of MRJ. These results are supported by the transmission electron micrograph data presented in this study, which revealed improvement in the ultrastructural integrity of ovarian tissues, reduction in follicular degeneration, and restoration of granulosa cell. The potential effect of methanolic extract of marjoram might be attributed to the reduction in cyst numbers and lipid droplets in the ovaries. Our result is in agreement with that of a previous study that reported the ability of marjoram to reduce body weight and ovary weight in the DHEA-induced PCOS rat model [21]. In contrast, the study of Haj-Husein et al. [39] reported that there was no significant effect of marjoram tea on the body weight of women with PCOS.

An abnormal lipid profile including raised LDL and triglyceride levels and decreased HDL levels is associated with PCOS [53]. Our data showed a remarkable increase in the serum levels of cholesterol, TGs, and LDL-c and a decrease in the serum levels of high-density lipoprotein (HDL) in rats treated with letrozole. In line with these results, a previous study reported a considerable decrease in HDL-c levels and an increase in triglyceride, cholesterol, and LDL-c levels in the letrozole group [54]. Interestingly, all of these lipid levels were reversed significantly with Met and MRJ treatments compared to letrozole treatment. This result can be attributed to the presence of phenolic compounds in marjoram, which may activate peroxisome proliferator-activated receptor alpha (PPARα), a receptor known for its role in the treatment of dyslipidemia [55].

One of the pathophysiological mechanisms that influences PCOS is IR, which in turn is accompanied by hyperinsulinemia in women with PCOS [56,57]. Previous studies showed that IR is a key pathological feature of PCOS in women [58,59]. However, the underlying mechanism of IR in PCOS remains unclear and needs to be elucidated, although hyperinsulinemia plays a vital role in PCOS-induced IR, resulting in anovulation and impaired follicular development. Indeed, an imbalance of steroid hormones is central to PCOS pathogenesis, featuring insulin resistance, low-grade inflammation, increased androgen levels, and OS [14,60,61,62]. Most women with PCOS have an elevated level of testosterone, which is a major hormone contributing to the pathogenesis of PCOS, and increased LH levels [63]. Similarly, in letrozole-induced rat models, we showed that both serum testosterone and LH levels were significantly increased in letrozole-treated rats, similar to hyperandrogenemia and follicular-phase estrogen levels of women with PCOS. Moreover, an alteration in the ratio of follicle-stimulating hormone (FSH) and LH in response to insulin has been observed, and an increase in the concentration of LH results in the downregulation of FSH [64]. Similarly, in the letrozole-induced PCOS rat model, the rats present a hyper-androgenized state characterized by the elevation in testosterone and LH levels [65,66,67]. Our findings revealed that, in contrast to the control group, the letrozole-treated rats showed significantly higher serum testosterone and LH levels and lower serum FSH and estradiol levels. These hormonal changes were reversed following treatment with metformin and all tested doses of MRJ. Numerous studies reported that metformin has a beneficial role in the improvement of the hormonal profile by exerting its effect on serum levels of insulin [14,68,69,70]. In this study, the homeostasis model of insulin resistance (HOMA-IR), as a marker of insulin resistance (IR), has been assessed according to fasting blood glucose and insulin levels. In this study, the metformin administration commendably reduced elevated markers emphasizing its potential to alleviate the metabolic disturbances caused by letrozole, but *Origanum majorana* L. did not significantly affect these metabolic parameters altered by letrozole, representing inadequate strength to change the outcomes, which is in contrast with a previous study reporting the beneficial effects of herbal treatments like marjoram on metabolic parameters in PCOS and other disorders [21,39]. A study by Rababa’h et al. [21] also showed that the marjoram-treated group had a significant effect on improving insulin sensitivity. Moreover, marjoram tea has shown a beneficial effect on the hormonal profile of women with PCOS by improving insulin sensitivity and by reducing the levels of adrenal androgens [38]. This discrepancy may arise from variances in study design factors, such as sample size, dosage, and treatment duration, and intervention methods.

In addition to the previously recognized factors involved in the pathophysiology of PCOS, the role of inflammation and OS in PCOS pathogenicity has been evaluated in the current study. Accumulated studies highlight the role of OS along with inflammation in the development of PCOS [71,72]. OS is a pathological state resulting from an imbalance between oxidants and antioxidants, which leads to an accumulation of reactive oxygen species (ROS) that act as a major factor in the pathogenesis of PCOS [73,74,75,76]. ROS exhibit diverse actions on cell function by regulating ion channels. Increased levels of ROS can increase Ca^2+^ ion levels from either endoplasmic reticulum or other stores, leading to a loss of intracellular Ca^2+^ homeostasis and affecting mitochondrial permeability [77,78]. A study by Rashidi et al. (2009) [79] reported that calcium deregulation causes follicular arrest in women with PCOS.

The activation of the intrinsic mitochondrial pathway of apoptosis is induced via the elevation in ROS levels, increased expression of Bax, release of cytochrome-c, activation of caspases 3 and 9, and downregulation of Bcl-2 [80]. Supporting the previous findings, our study showed that letrozole raised Bax and caspase-3 levels and lowered Bcl-2 levels in the ovaries. However, treatment with metformin or MRJ at 20 and 40 mg/kg showed partial improvement in PCOS symptoms. In addition, insufficient antioxidant levels caused the augmented production ROS, which might be another factor that contributes to the development of PCOS [81,82]. Antioxidants have a vital role in reducing the destructive effect of free radicals [72]. In addition to regulating redox stress, Nrf2 also regulates the antioxidant gene expression by binding to the antioxidant response element [83,84]. It is a potent antioxidant as it induces the transcription of antioxidant genes. In this regard, the activation of Nrf2 signaling is considered as one of the most important signaling pathways that is targeted by therapeutic strategies for reducing OS in patients with polycystic ovary syndrome [85]. In addition to its antioxidant properties, it also acts as an anti-apoptotic and anti-inflammatory agent by inhibiting NF-κB activation and upregulating Bcl-2 expression [86,87,88,89]. OS and inflammation are linked processes. The crosstalk between the two signaling pathways of Nrf2 and NF-κB is highly complex and complicated and involves multiple regulatory mechanisms. Nrf2 decreases NF-κB activation whereas NF-kB inhibits the activation of Nrf2 at the transcription level [90]. Hence, reactive oxygen species (ROS) play a vital role in inducing inflammation by activating NF-kB and increasing the levels of pro-inflammatory cytokines [91,92]. As suggest by Artimani et al. [93], there is a significant link between the levels of TNF-α and the levels of OS in PCOS patients, reflecting its role in the progression of inflammation and OS by stimulating the secretion of pro-inflammatory cytokines. In line with earlier reports, PCOS ovaries experienced OS, demonstrated by the rise in pro-inflammatory biomarkers, in which the reduction in the levels of all these pro-inflammatory factors was progressively and significantly decreased with the increase in MRJ dose compared to letrozole-treated rats. The antioxidant activity of MRJ was demonstrated in the current study by a substantial elevation in antioxidant enzyme levels (GSH, SOD, and HO-1) in the letrozole-treated rat’s ovarian homogenates. Increasing levels of GSH in the ovaries of letrozole-treated rats that received increasing doses of MRJ (20, 40, and 60 mg/kg) suggest that this effect may result from the enhanced biosynthesis of GSH or reduced OS, which contribute to low degradation of GSH or both effects. Additionally, one of the most powerful antioxidants is SOD, which catalyzes the formation of hydrogen peroxide from superoxide radicals, thus diminishing OS [94,95]. Similarly, Li et al. [96] also reported that marjoram is rich in phytochemical constituents and flavonoids, which possess antioxidant and anti-inflammatory activities.

Based on the results obtained in this study, *Origanum majorana* L. extract showed beneficial effects on the hormonal profile, OS, and inflammation, suggesting its potential therapeutic effect for PCOS patients. The animal (rat) dose of MRJ (20, 40, and 60 mg/kg) corresponds to the human equivalent dose (HED) of approximately 3.23, 6.45, and 9.68 mg/kg and is calculated according to the conversion relationship of the Food Drug Administration [97]. These values provide a basis for designing early-phase clinical trials with an appropriate safety margin, and further preclinical studies, including toxicity, pharmacokinetics, and formulation development, are necessary before progressing to clinical trials.

This study presents both significant strengths and notable limitations. It has been identified that marjoram contains bioactive compounds that are anti-inflammatory, antibacterial, and antioxidant, which demonstrate its potential as a valuable addition to holistic and plant-based therapies for treating PCOS and its associated metabolic disorders. It has been noted that marjoram reduces OS markers and lipid profiles in PCOS patients, suggesting its potential inclusion in dietary and therapeutic regimens. One of the study limitations is the short duration of the study, and a longer treatment duration may be necessary to assess sustained effects, particularly in terms of long-term hormonal regulation, ovarian function, and metabolic outcomes. This study focused on metabolic and hormonal markers and found that marjoram did not significantly impact insulin or glucose levels, possibly due to the dosage or duration used, thus emphasizing the need for further research using various dosages and extended treatment periods. It is important to note that, while animal studies provide valuable insights, there is still a need to confirm the efficacy and safety of Origanum in women with PCOS.

## 4. Materials and Methods

### 4.1. Animals

Forty-two adult healthy female Wistar albino rats (8 weeks old, 170 ± 15 g) were obtained from the Experimental Animal Care Centre, King Saud University, Riyadh, Saudi Arabia. Rats were acclimated for 1 week before starting the experiment. All animals were housed in stainless steel cages (seven rats/cages) in an air-conditioned room (12 h light/12 h dark cycle) at 21–23 °C and 50 ± 10% of relative humidity during the adaptation period (1 week) and throughout the experimental period, and they were fed with a standard laboratory diet and water ad libitum.

### 4.2. Preparation of Methanolic Marjoram Extract

Marjoram (*Origanum majorana* L.) (MRJ) dried leaves were purchased from a certified local supplier in Riyadh, Saudi Arabia, during its cultivation period. The source and producer is Al-Manar Corporation, Egypt, and the importer is Osoul Al-Izdihar Trading Corporation, Saudi Arabia. The methanolic extract of MRJ dried leaves was prepared as previously described in our study [98]. In brief, the dried leaves of MRJ were blended to yield a powder, and after blending, the powder (500 g) was extracted for 24 h at room temperature on a shaker in a sterile bottle containing 1 L of methanol (Sigma, St. Louis, MO, USA), and the extraction was repeated thrice. Afterward, the extract was filtered using a Whatman filter (Whatman, Clifton, NJ, USA), and the solvent was removed by reducing the pressure using a vacuum pump. The dry extract was collected and kept at 4 °C in a refrigerator until further use.

### 4.3. Quantitative Analysis of Major Compounds in Origanum majorana L.

The methanolic *Origanum majorana* L. dried leaves extract was injected into a silica capillary column (30 m × 0.25 mm I.D. × 0.25 μm film thickness) of the GC-MS instrument (Agilent 6890N/5973I, California, CA, USA) with a mass selective detector to detect its chemical composition. The instrument settings were as follows:

Temperature—an initial temperature of 70 °C, holding for 2 min, increased to 305 °C at 20 °C/min, followed by holding for 1 min.

Total GC running time: 45 min.

Carrier gas: helium gas (99.999%).

Flow rate: 1.2 mL/min.

Injector temperature: 250 °C.

Ion-source temperature: 230 °C.

Based on the GC-MS spectrum, the relative percentage of the corresponding component was calculated, and the mass spectra of the unknown compounds were identified by comparing them with the known 62,000 patterns available in the National Institute of Standard and Technology (NIST08) computer library [98].

### 4.4. Drugs

Letrozole (Cat. No. L6545) and metformin (Cat. No. 317240) powders were purchased from Sigma Aldrich, St. Louis, MO, USA. Both drugs as well as the MRJ dry extract were freshly dissolved in 0.5% carboxymethylcellulose (CMC) (Cat. No. C5678, Sigma Aldrich, St. Louis, MO, USA).

### 4.5. Experimental Design

In the present study, 42 rats were divided into 6 groups (7 in each group) as follows: (1) the control group: administered 0.5% CMC as a vehicle; (2) the letrozole-treated group: treated with letrozole solution (1 mg/kg/day); (3) the letrozole + metformin-treated group: administered letrozole and co-treated with metformin solution (2 mg/100 g/day); (4) the letrozole + MRJ (20 mg/kg)-treated group: administered letrozole and co-treated with MRJ extract solution (20 mg/kg/day); (5) the letrozole + MRJ (40 mg/kg)-treated group: administered letrozole and co-treated with MRJ extract solution (40 mg/kg/day); and (6) the letrozole + MRJ (60 mg/kg)-treated group: administered letrozole and co-treated with MRJ extract solution (60 mg/kg/day). All treatments were given via the mouth (p.o.) for a total period of 21 days.

### 4.6. Dose Selection

The dose of letrozole (1 mg/kg, per orally, p.o.) was adopted from previous studies [34,99,100,101] that have confirmed the development of PCOS and IR and the induction of ovarian damage via the activation of inflammation and OS. As in the Rababa’h et al. [21] study, a 21-day treatment period was chosen to assess the therapeutic effects of marjoram extract and the possibility of reversing PCOS-related alterations. The dose of metformin as a positive protective control to alleviate oxidative and inflammatory ovarian damage in letrozole-treated female rats was selected in accordance with the studies of Ibrahim et al. (2022) [54] and Ibrahim et al. [99]. The minimum dose of 20 mg/kg was chosen based on the study by Rababa’h et al. [21] that demonstrated the initial therapeutic potential of the marjoram extract at this dose, while the doses of 40 mg/kg and 60 mg/kg were guided by the work of Elfiky et al. [36] who investigated low (50 mg/kg) and high (100 mg/kg) doses of marjoram extract in a DHEA-induced PCOS rat model. These studies have shown partial antioxidant and anti-inflammatory protective effects of MRJ against dehydroepiandrosterone (DHEA)-induced PCOS model in female rats. However, in our preliminary data, we have shown a dose antioxidant protective effect of MRJ in letrozole-treated ovaries with the maximum similar effect seen at doses between 60 and 100 mg/kg.

### 4.7. Tissue and Blood Collection

At the end of the experimental period, all rats were anesthetized with 90 mg/kg of ketamine hydrochloride and 10 mg/kg of xylazine hydrochloride [102]. Around 1 mL of blood from each rat was collected in EDTA-containing or plain tubes and centrifuged (500× *g* for 15 min) to collect plasma and serum, respectively, which were stored at −20 °C until further use.

Further, cervical dislocations ethically killed the animals, and their abdomens were opened to localize the ovaries. Following identification, the ovaries were collected on ice, weighed, and sectioned into smaller pieces, with portions fixed in 2.5% glutaraldehyde for electron microscopy, and the remaining parts were snap-frozen in liquid nitrogen and stored at −80 °C.

Remaining frozen ovaries were homogenized in ice-cold phosphate-buffered saline (PBS) (pH = 7.4) and then centrifuged for 20 min at 11,400× *g* at 4 °C. Finally, all supernatants (tissue homogenates) were collected and kept at −80 °C until further use.

### 4.8. Measurements in the Plasma and the Serum

Plasma glucose (Cat. No. 81695, Chrystal Chem, Elk Grove Village, IL, USA) and insulin levels (Cat. No. ERINS, ThermoFisher, Waltham, MA, USA) were measured using ELISA kits. The levels of IR, as measured by the homeostasis model of insulin resistance (HOMA-IR), were calculated as mentioned previously [103]:

HOMA-IR = [fasting glucose (mg/dL) × fasting insulin (µIU/mL)]/405.

Serum levels of testosterone (Cat. No. 80550; Chrystal Chem, Elk Grove Village, IL, USA), FSH (Cat. No. EA0015Ra, Bioassay Technology Laboratory, Shanghai, China), LH (Cat. No. E-EL-R0026; Elabscience, Houston, TX, USA), and estradiol (Cat. No. 80548 Chrystal Chem, Elk Grove Village, IL, USA) were measured using ELISA kits. Serum levels of total cholesterol (CHOL), total triglycerides (TGs), low-density lipoprotein-cholesterol (LDL-c), and high-density lipoprotein-cholesterol (HDL-c) were measured using assay kits (Cat. No. 10009582, Cayman Chemicals, Ann Arbor, MI, USA; Cat. No. ECCH-100, BioAssay Systems, Hayward, CA, USA; Cat. No. 79960 and Cat. No. 79970, Crystal Chemicals, Houston, TX, USA, respectively). All kits used in this study were rat-specific, and all measurements in the plasma and serum were performed in duplicate for n = 7 rats/group.

### 4.9. Biochemical Analysis in the Tissue Homogenates

Levels of SOD, MDA, total glutathione (GSH), and heme-oxygenase-1 in the ovarian homogenates were measured using ELISA kits (Cat. No. RTFI00215, Assay Genie, London, UK; Cat. No. MBS268427, MyBioSource, San Diego, CA, US; Cat. No. RTEB0206 Assay Genie, London, UK; and Cat. No. MBS764989, MyBioSource, San Diego, CA, USA, respectively). Similarly, the ovarian levels of IL-6, TNF-α, ICAM-1, and TLR-4 were also measured by ELISA kits (Cat. No. MBS2507393; Cat. No. MBS1600418, MyBioSource, San Diego, CA, USA; Cat. No. CSB-E04576, CUSABIO, Houston, TX, USA, and Cat. No. MBS705488 MyBioSource, San Diego, CA, USA; respectively). The levels of markers of intrinsic cell apoptosis including Bcl-2, Bax, and caspase-3 in the ovarian homogenates were determined by ELISA kits (Cat. No. MBS2881713, Cat. No. MBS935667 and Cat. No. MBS018987 MyBiosources, San Diego, CA, USA, respectively).

### 4.10. Preparation of Cytoplasmic and Nuclear Fractions and Analysis

A nuclear/cytoplasmic extraction kit was used to prepare the cytoplasmic and nuclear fractions from the frozen ovaries (Cat. No. 4110147; Bio-Rad, Hercules, CA, USA). The cytoplasmic and nuclear levels of Nrf2 and NF-κB in the cytoplasmic and nuclear extracts were measured using Nrf2, and fractions were assessed by rat-specific ELISA kits (Cat # MBS752046 and Cat MBS453975, My Biosources, San Diego, CA, USA).

#### Real-Time Polymerase Chain Reaction (q-PCR)

The primer pair sequences of Nrf2, NF-κB, and β-actin used for the q-PCR reaction have been reported in earlier studies [104]. Total RNA was extracted using a commercial kit (cat 74004; Qiagen, Hilden, Germany), while the first-strand cDNA was synthesized using a cDNA synthesis commercial kit (cat K1621, The Thromo Fisher kit, Waltham, MA, USA). The amplification reaction was performed in a CFX96 PCR machine using the Ssofast Evergreen Supermix Kit (cat 172-5200, Bio-Rad, Hercules, CA, USA). The amplification reactions were set as follows:

(1) Heating: 1 cycle/98 °C/30 s;
(2) Denaturation: 40 cycles/98 °C/5 s;
(3) Annealing: 40 cycles/60 °C/5 s;
(4) Melting: 1 cycle/95 °C/5 s/step.

The relative mRNA expression of all target genes was presented after the normalization of GAPDH using the 2^ΔΔC^_T_ method.

### 4.11. Ultrastructural Study

Freshly collected ovary specimens (2–3 mm^3^; n = 7) were fixed in a glutaraldehyde solution (2.5%) and prepared in a sodium cacodylate buffer (0.1 M, pH 7.2) for 6 h in the fridge. All samples were then exposed to 1% osmium tetroxide solution post-fixation. After that, samples were dehydrated in an ascending series of ethanol, followed by embedding in the Spurr’s resin. All specimens were segmented at a thickness of 0.5 μm and then were stained with toluidine blue, followed by uranyl acetate and lead citrate stains. The examination was conducted and photographs were taken by a blinded pathologist at the electron microscopy unit of the College of Medicine at King Saud University using an electron microscope (model JEM-101) (Jeol Co., Tokyo, Japan).

### 4.12. Statistical Analysis

All data were analyzed using one-way ANOVA with the GraphPad Prism software (version 8). Normality was assessed using the Kolmogorov–Smirnov test, and group comparisons were performed using Tukey’s post hoc test. Differences were considered statistically significant at *p* < 0.05.

## 5. Conclusions

The current study highlights a protective effect of marjoram against letrozole-induced ovarian damage in rats with polycystic ovarian syndrome, and it can be used as a therapeutic agent in preventing and treating PCOS. Moreover, this protective effect is associated with hypolipidemic, antiapoptotic, antioxidant, and anti-inflammatory effects. The mechanisms underlying the protective effects of marjoram are mediated by the stimulation of the Nrf2 signaling pathway and the suppression of NF-κB.

## Figures and Tables

**Table 1:** Quantitative analysis of marjoram methanolic extract.

Sr. No	Retention Time (min)	Area (m^2^)	Name	Molecular Weight	Percentage (%)
1	5.20	72,500	5-Hydroxymethylfurfural	126	2.28
2	12.35	62,500	Dodecanoic acid	200	1.96
3	18.60	107,000	Deca-4,6-diyn-1-yl 3-methylbutanoate	234	3.36
4	19	98,000	(*E*)-Deca-8-en-4,6-diyn-1-yl 3-methylbutanoate	232	3.08
5	19.15	65,000	3,7,11,15-Tetramethyl-2-hexadecen-1-ol	296	2.04
6	19.25	67,000	2-Pentadecanone, 6,10,14-trimethyl-	268	2.10
7	21.25	73,000	Hexadecanoic acid, methyl ester	270	2.29
8	22.5	115,000	*n*-Hexadecanoic acid	256	3.61
9	24.90	74,500	7,10-Octadecadienoic acid, methyl ester	294	2.34
10	25	74,000	9,12,15-Octadecatrienoic acid, ethyl ester, (*Z*,*Z*,*Z*)-	306	2.32
11	25.25	66,000	[1,1-Bicyclopropyl]-2-octanoic acid, 2′-hexyl-, methyl ester	322	2.07
12	25.85	125,000	Linoleyl acetate	308	3.93
13	26	139,000	9,12,15-Octadecatrienoic acid, (*Z*,*Z*,*Z*)	278	4.37
14	26.30	112,000	Octadecanoic acid	284	3.52
15	26.60	78,000	Cyclopropanetetradecanoic acid, 2-octyl-, methyl ester	394	2.45
16	27.40	66,000	d-Mannitol, 1-*O*-(22-hydroxydocosyl)-	506	2.07
17	27.55	73,000	1b,4a-Epoxy-2*H*-cyclopenta[3,4]cyclopropa[8,9]cycloundec[1,2-*b*]oxiren-5(6H)-one, 7-(acetyloxy)decahydro-2,9,10-trihydroxy-3,6,8,8,10a-pentamethyl-	424	2.29
18	28.20	116,000	Tetrapentacontane	758	3.64
19	29	95,000	Eicosanoic acid	312	2.98
20	29.10	150,000	Henicosane-6,8-dione	324	4.71
21	30.6	91,000	Hexadecanoic acid, 2-hydroxy-1-(hydroxymethyl)ethyl ester	330	2.86
22	30.75	76,000	Butylaldehyde, 4-benzyloxy-4-[2,2,-dimethyl-4-dioxolanyl]-	278	2.39
23	32.45	85,000	Linoleyl myristate	476	2.67
24	32.55	94,000	9,12,15-Octadecatrienoic acid, 2,3-dihydroxypropyl ester, (*Z*,*Z*,*Z*)	352	2.95
25	33.35	105,000	Pentacosane-6,8-dione	380	3.30
26	33.45	90,000	Tricosane-4,6-dione	352	2.83
27	33.65	78,000	2,2,4-Trimethyl-3-(3,8,12,16-tetramethyl-heptadeca-3,7,11,15-tetraenyl)-cyclohexan-1-ol	428	2.45
28	35.30	142,000	Heptacosane-6,8-dione	408	4.46
29	36.85	106,000	DL-alpha-Tocopherol	430	3.33
30	37.75	102,000	Nonacosane-6,8-dione	436	3.20
31	38.65	120,000	Stigmasterol	412	3.77
32	39.05	151,000	1,1,6-Trimethyl-3-methylene-2-(3,6,9,13-tetramethyl-6-ethenye-10,14-dimethylene-pentadec-4-enyl)cyclohexane	452	4.74
33	39.70	115,000	gamma-Sitosterol	414	3.61

**Table 2:** Final body weight and final ovary weights and lengths in all experimental groups.

	Final Body Weight (g)	Right Ovary Weight (mg)	Left Ovary Weight (mg)	Right Ovary Length (mg)	Left Ovary Length (mg)
Control	232.1 ± 24.5	46.4 ± 4.7	47.2 ± 3.2	4.5 ± 0.4	4.7 ± 0.3
Letrozole	364.5± 29.4 ^a^	65.4 ± 6.1 ^a^	66.0 ± 6.0 ^a^	6.4 ± 0.51 ^a^	6.8 ± 0.6 ^a^
Letrozole + Met	284.3± 14.5 ^ab^	57.2 ± 4.2 ^ab^	55.5 ± 3.8 ^ab^	5.8 ± 0.3 ^ab^	5.9 ± 0.5 ^ab^
Letrozole+ MRJ (20 mg/kg)	367.5± 27.8 ^ac^	57.8 ± 5.8 ^ab^	57.5 ± 3.8 ^ab^	5.7 ± 0.4 ^ab^	5.7 ± 0.3 ^ab^
Letrozole + MRJ (40 mg/kg)	381.3 ± 34.5 ^ac^	50.8 ± 4.1 ^abcd^	52.3 ± 2.5 ^abcd^	5.1 ± 0.3 ^abcd^	5.2 ± 0.3 ^abcd^
Letrozole + MRJ (60 mg/kg)	378.1 ± 40.1 ^ac^	43.7 ± 3.2 ^bcde^	46.6 ± 3.0 ^bcde^	4.2 ± 0.4 ^bcde^	4.6 ± 0.5 ^bcde^

Data are presented as means ± SD for n = 7 rats/group. Values were considered significantly different at *p* < 0.05. (a): Significantly differed from the control group; (b): significantly differed from the letrozole-treated rats; (c): significantly differed from the letrozole + metformin (Met)-treated rats; (d): significantly differed from the letrozole + MRJ-treated animals (20 mg/kg); and (e): significantly differed from the letrozole + MRJ-treated animals (40 mg/kg).

**Table 3:** Fasting blood glucose (FBG), fasting blood insulin (FBI), and HOMA-IR in all experimental groups of rats.

	FBG (mg/dL)	FBI (mIU/mL)	HOMA-IR
Control	98.5 ± 10.1	4.1 ± 0.73	0.93 ± 0.11
Letrozole (PCOS)	171.3 ± 19.4 ^a^	6.9 ± 0.61 ^a^	2.91± 0.31 ^a^
Letrozole + Met	131.3 ± 14.3 ^ab^	4.9 ± 0.6 ^ab^	1.68 ± 0.21 ^ab^
Letrozole + MRJ (20 mg/kg)	169.1 ± 17.5 ^ac^	6.3 ± 0.6 ^ac^	2.52 ± 0.45 ^ac^
Letrozole + MRJ (40 mg/kg)	184.3 ± 20.1 ^ac^	6.1 ± 0.6 ^ac^	2.71 ± 0.71 ^ac^
Letrozole+ MRJ (60 mg/kg)	177.3 ± 16.5 ^ac^	6.5± 0.7 ^ac^	2.88 ± 0.41 ^ac^

Data are presented as means ± SD for n = 7 rats/group. Values were considered significantly different at *p* < 0.05. (a): Significantly differed from the control group; (b): significantly differed from letrozole-treated rats; (c): significantly differed from letrozole + metformin (Met)-treated rats.

**Table 4:** Serum lipid profile of all groups of rats.

	CHOL (mg/dL)	TGs (mg/dL)	LDL-c (mg/dL)	HDL-c (mg/dL)
Control	68.9 ± 9.1	88.5 ± 9.9	34.5 ± 3.1	20.1± 2.3
Letrozole (PCOS)	181.2 ± 20.1 ^a^	144.3 ± 12.1 ^a^	102.2 ± 9.5 ^a^	8.5 ± 0.79 ^a^
PCOS + met	137.2 ± 14.6 ^ab^	119.3 ± 10.3 ^ab^	76.5 ± 5.5 ^ab^	15.4 ± 1.9 ^ab^
PCOS + MRJ (20 mg/kg)	146.3 ± 12.1 ^abc^	124.5 ± 10.4 ^ab^	78.4 ± 7.8 ^ab^	12.5 ± 1.1 ^ab^
PCOS + MRJ (40 mg/kg)	118.3 ± 11.6 ^abcd^	102.1 ± 8.5 ^abcd^	64.3 ± 5.1 ^abcd^	16.5 ± 1.2 ^abcd^
PCOS + MRJ (60 mg/kg)	81.3 ± 8.5 ^abcde^	83.5 ± 6.9 ^bcde^	39.6 ± 4.7 ^bcde^	22.4 ± 1.8 ^bcde^

Data are presented as means ± SD for n = 7 rats/group. Values were considered significantly different at *p* < 0.05. (a): Significantly differed from the control group; (b): significantly differed from letrozole-treated rats; (c): significantly differed from letrozole + metformin (Met)-treated rats; (d): significantly differed from letrozole + MRJ-treated animals (20 mg/kg); and (e): significantly differed from letrozole + MRJ-treated animals (40 mg/kg). Note: CHOL: cholesterol; TGs: triglycerides; LDL-c: low-density lipoprotein-cholesterol; HDL-c: high-density lipoprotein-cholesterol.

## Data Availability

The original contributions presented in this study are included in the article. Further inquiries can be directed to the corresponding author.

## Abbreviations

The following abbreviations are used in this manuscript:

Bax	Bcl-2-associated protein x
Bcl-2	B-cell lymphoma 2
CHOL	total cholesterol
FBG	fasting blood glucose
FBI	fasting blood insulin
FSH	Follicular-stimulating hormone
GSH	total glutathione
HDL	high-density lipoprotein-cholesterol
HO-1	heme-oxygenase-1
HOMA-IR	homeostasis model of insulin resistance
ICAM-1	intracellular cell adhesion molecule-1
IL-18	interleukin-18
IL-1β	interleukin-1 beta
IL-6	interleukin-6
IR	insulin resistance
LDL-c	low-density lipoprotein-cholesterol
LH	luteinizing hormone
MDA	malondialdehyde
Met	metformin
MRJ	marjoram (*Origanum majorana* L.)
mRNA	messenger RNA
NF-κB	nuclear factor kappa B
Nrf2	nuclear factor erythroid 2-related factor 2
OS	oxidative stress
PCOS	polycystic ovary syndrome
ROS	reactive oxygen species
SOD	superoxide dismutase
T2D	type 2 diabetes
TGs	total triglycerides
TLR4	toll-like receptor
TNF-α	tumor necrosis factor-α
